# Bioinformatics-led discovery of liver-specific genes and macrophage infiltration in acute liver injury

**DOI:** 10.3389/fimmu.2023.1287136

**Published:** 2023-12-07

**Authors:** Zhiwen Cao, Peipei Lu, Li Li, Qi Geng, Lin Lin, Lan Yan, Lulu Zhang, Changqi Shi, Li Li, Ning Zhao, Xiaojuan He, Yong Tan, Cheng Lu

**Affiliations:** Institute of Basic Research in Clinical Medicine, China Academy of Chinese Medical Sciences, Beijing, China

**Keywords:** acute liver injury, immunoinfiltration, bioinformatics, acetaminophen, triptolide

## Abstract

**Background:**

Acute liver injury (ALI) is an important global health concern, primarily caused by widespread hepatocyte cell death, coupled with a complex immune response and a lack of effective remedies. This study explores the underlying mechanisms, immune infiltration patterns, and potential targets for intervention and treatment ALI.

**Methods:**

The datasets of acetaminophen (APAP), carbon tetrachloride (CCl4), and lipopolysaccharide (LPS)-induced ALI were obtained from the GEO database. Differentially expressed genes (DEGs) were individually identified using the limma packages. Functional enrichment analysis was performed using KEGG, GO, and GSEA methods. The overlapping genes were extracted from the three datasets, and hub genes were identified using MCODE and CytoHubba algorithms. Additionally, PPI networks were constructed based on the String database. Immune cell infiltration analysis was conducted using ImmuCellAI, and the correlation between hub genes and immune cells was determined using the Spearman method. The relationship between hub genes, immune cells, and biochemical indicators of liver function (ALT, AST) was validated using APAP and triptolide (TP) -induced ALI mouse models.

**Results:**

Functional enrichment analysis indicated that all three ALI models were enriched in pathways linked to fatty acid metabolism, drug metabolism, inflammatory response, and immune regulation. Immune analysis revealed a significant rise in macrophage infiltration. A total of 79 overlapping genes were obtained, and 10 hub genes were identified that were consistent with the results of the biological information analysis after screening and validation. Among them, Clec4n, Ms4a6d, and Lilrb4 exhibited strong associations with macrophage infiltration and ALI.

## 1 Introduction

The liver, a vital metabolic organ in the human body, is primarily responsible for various physiological functions, including metabolism, detoxification, and protein synthesis. Unfortunately, liver damage often occurs due to factors such as drug abuse and alcohol consumption. Acute liver injury (ALI) is a condition characterized by dysfunction in innate immunity and damage to liver cell, and its high mortality rate has attracted clinical attention (1, 2). The mechanisms involved in ALI are intricate, encompassing multiple signaling pathways, including oxidative stress, inflammatory responses, and immune reactions, among others (3, 4). Although there is a deep understanding of the pathological features of ALI, the molecular characteristics that induce ALI is still not thorough enough (5). ALI progresses rapidly, and currently, there are no drugs available to reverse this deteriorative process (5, 6). Therefore, exploring the general mechanisms and targets associated with ALI is of great significance in formulating effective treatment strategies.

The diverse factors responsible for local sterile inflammation within innate immunity are the primary culprits of liver injury and failure (7). In various ALI models, immune cell infiltration in the liver, including neutrophils, NK/NKT cells, T cells, and macrophages, represents a critical pathological feature, each playing distinct roles (8–12). For example, the activation of macrophages during ALI can lead to the release of reactive oxygen species, IL-6, and TNF-α, thereby inducing apoptosis of liver cells (13, 14). In the case of APAP-induced ALI, eosinophils can be recruited to the liver, exerting hepatoprotective effects (15). Therefore, understanding the immune response in ALI and identifying its potential regulatory targets is paramount.

Bioinformatics provides an efficient method to predict potential regulatory targets and associated mechanisms for diseases. It may be a new attempt to obtain the general regulatory characteristics of ALI by combining the open database to obtain the data sets of different liver injury models as predictive models, which may be a new attempt to explore potential strategies for addressing ALI.

**Figure 1:**
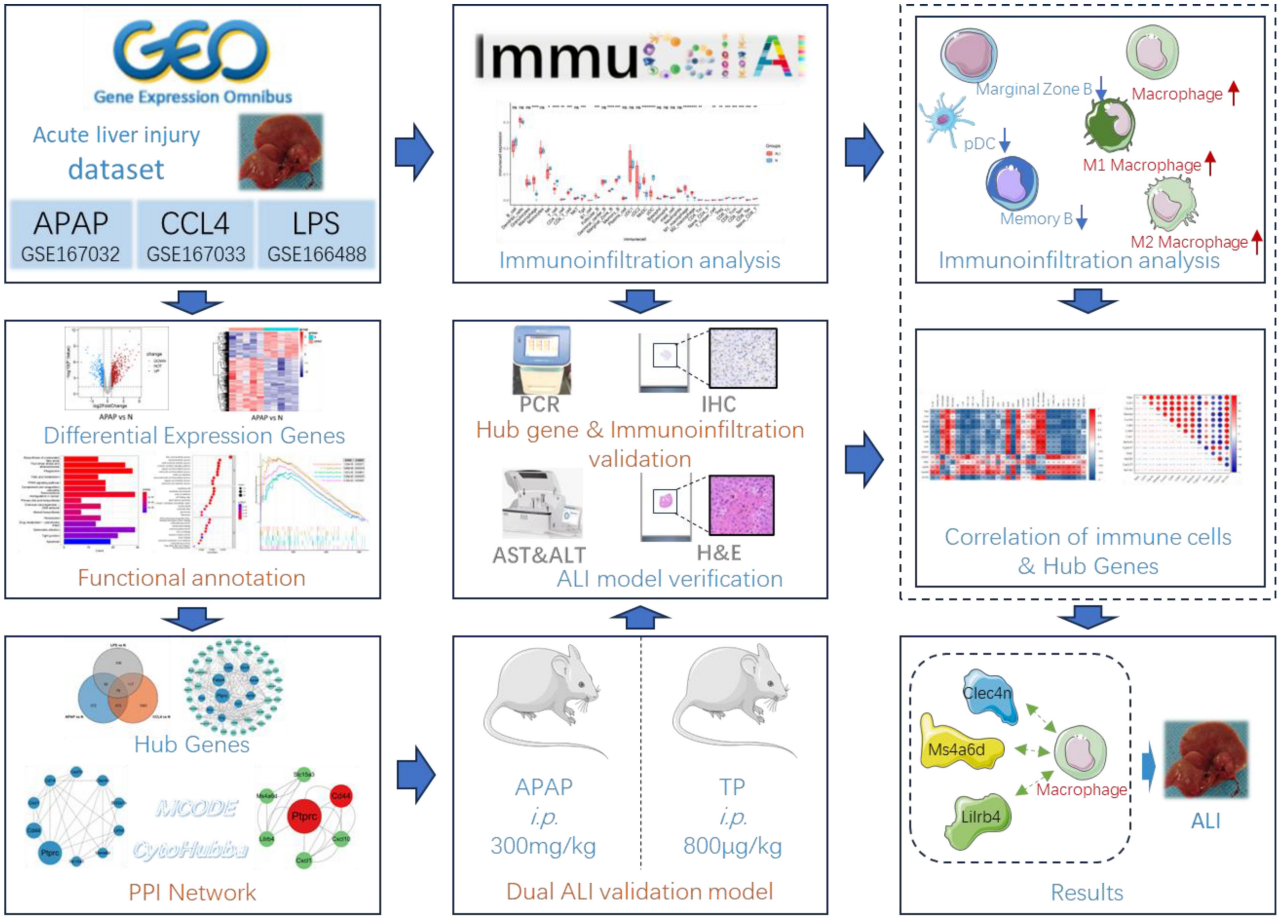
Flow chart of bioinformatics analysis and animal experiments. The figure was partly generated using Servier Medical Art, provided by Servier, licensed under a Creative Commons Attribution 3.0 unported license.

In light of this, the present study employed a dataset encompassing acetaminophen (APAP), carbon tetrachloride (CCl4), and lipopolysaccharide (LPS)-induced ALI to investigate potential pathways, immune response mechanisms, and regulatory targets of ALI. The APAP and triptolide (TP)-induced ALI models were selected for validation of the predictive results. The ALI models induced by APAP, CCl4, and LPS have been widely used for exploring the mechanisms of ALI and validating drug efficacy. Previous studies have indicated that these models cover mechanisms of inflammation response, mitochondrial function, oxidative stress, immune regulation, apoptosis, necrosis, and more (1, 5, 16, 17). In clinical practice, the overdose or long-term abuse of APAP can deplete glutathione and lead to the accumulation of its metabolite, N-acetyl-p-benzoquinone imine (NAPQI), resulting in ALI. TP, the primary active component of Tripterygium wilfordii Hook.f, exhibits multi-organ toxicity, particularly hepatotoxicity, which limits its clinical application. The mechanisms underlying TP-induced ALI are associated with oxidative stress and immune imbalance (18–20). Through these three predictive datasets and two validated animal models, this study aims to explore the general gene regulatory profiles and immune infiltration characteristics in the progression of ALI (**Figure 1**). Such investigations will contribute to a deeper understanding of the pathogenesis of ALI and may pave the way for developing novel therapeutic strategies.

## 2 Methods

### 2.1 Microarray data source

The datasets GSE167032, GSE167033, and GSE166488 were obtained from the GEO database (https://www.ncbi.nlm.nih.gov/geo/) (21). GSE167032 ([Mouse430_2] Affymetrix Mouse Genome 430 2.0 Array) is a mouse ALI model induced by a single injection of 300 mg/kg APAP, lasting 24 hours. This dataset comprises 5 control samples and 5 APAP-treated samples. The gene expression data of GSE167033 ([Mouse430_2] Affymetrix Mouse Genome 430 2.0 Array) consists of 10 samples obtained from mice treated with a single injection of 1.6 g/kg CCl4. These samples were collected at the 24-hour time point, including 5 samples from the control group and 5 samples from the model group. GSE166488 ([Mouse430_2] Affymetrix Mouse Genome 430 2.0 Array) is a mouse ALI model induced by a single injection of 750 μg/kg LPS for 24 hours. The dataset includes 5 samples from the model group and 3 samples from the control group.

### 2.2 Acquisition of microarray data and identifcation of DEGs

The three datasets were retrieved from the GEO database and processed using the limma package in R (22). Differential gene expression analysis was conducted on each dataset, applying a filtering criterion of |logFC| > 0.75 and a P. value < 0.05 to identify significant genes. The results of the differential gene analysis were visualized using volcano plots and heatmaps, created with the ggplot2 and pheatmap packages, respectively (23).

### 2.3 Functional enrichment analysis

Gene Ontology (GO) and Kyoto Encyclopedia of Genes and Genomes (KEGG) pathway enrichment analyses ware conducted using the R package clusterProfiler (24), with the DEGs criteria set as |logFC|>0.75 and P.Value<0.05. The resulting analysis outcomes ware then visualized using the gglot2 package (23). Additionally, GSEA analysis is performed using the clusterProfiler package with a pvalueCutoff of 0.05, and the obtained results ware further visualized using gglot2 for enhanced visualization and interpretation (23, 24).

### 2.4 Analysis of protein–protein interactions and identifcation of Hub genes

Perform protein–protein interactions (PPI) analysis on the overlapping DEGs from three datasets using the String database (https://cn.string-db.org/) and visualize the results using Cytoscape 3.9.1 (25). Utilize the MCODE and CytoHubba plugins to respectively filter hub genes, and integrate the obtained DEGs to form a hub gene set (26, 27). Using the MCODE plugin in Cytoscape, the advanced options ware set as follows: in Network Scoring, the Degree Cutoff was set to 2 (26). In Cluster Finding, the Node Score Cutoff was set to 0.2, the K-Core was set to 2, and the Max Depth was set to 100. Plugin CytoHubba selected the MCC algorithm (27).

### 2.5 Immune infltration analysis

Immunoinfiltration analysis was conducted using ImmuCellAI-mouse (http://bioinfo.life.hust.edu.cn/ImmuCellAI-mouse/#!/) (28, 29). Due to the lower gene count in dataset GSE167032 compared to the other two datasets, immunoinfiltration analysis was focused on GSE167033 and GSE166488 datasets. The GSE167033 and GSE166488 datasets, as well as their combined normalized dataset, were individually assessed for the degree of infiltration of 36 immune cell types using ImmuCellAI-mouse. The resulting datasets underwent inter-group differential analysis using Wilcox.test and were then visualized using ggplot2 (23). The correlation analysis and visualization of hub genes and immune cells were conducted using OriginPro.

### 2.6 Establishment of animal model of ALI

According to previous reports, TP and APAP ALI models were constructed to verify the expression of Hub genes and immune infiltration. Female Balb/c mice (6-8 weeks old) obtained from Beijing Vital River Laboratory Animal Technology Co., Ltd (SCXK (jing) 2021–0006). Animal experiments strictly adhered to the Guide for the Care and Use of Laboratory Animals, and the experimental protocol was approved by the Research Ethics Committee of the Institute of Basic Theory of Chinese Medicine, China Academy of Chinese Medical Sciences (IBTCMCACMS21-2110-04). After one week of acclimatization, the mice were randomly divided into control, TP, and APAP groups. The control group received an equivalent volume of physiological saline, the TP group received 800ug/kg of TP, and the APAP group received 300mg/kg of APAP. All administrations were done via intraperitoneal injection. After 24 hours of drug administration, all mice were euthanized and liver tissue and serum samples were collected for subsequent analysis.

### 2.7 Detection of ALT/AST level

The blood samples were collected in centrifuge tubes without anticoagulants, and serum was obtained by centrifugation. The levels of ALT and AST were detected using an automated biochemical analyzer.

### 2.8 RNA extraction and qRT−PCR

Liver tissue was homogenized, and RNA extraction was performed using the RNAsimple Total RNA Kit (Tiangen Biotech). RNA concentration was determined using NanoDrop2000, and reverse transcription was carried out using the First-Strand Synthesis Master Mix (Lablead Biotech). The qRT-PCR step was conducted using the SYBR Green PCR Fast mixture (Lablead Biotech), and the expression levels of the target genes were normalized to GAPDH. The primer sequences for all genes are provided in **Table S1**.

### 2.9 Immunohistochemistry

Paraffin-embedded sections were dewaxed to water, followed by antigen retrieval. A 3% hydrogen peroxide solution was added to block endogenous peroxidase activity, and 3% BSA was applied within the tissue area to block non-specific binding. The primary antibody was added and incubated overnight at 4°C. After washing, the sections were incubated with the secondary antibody. Subsequently, DAB staining was performed, followed by counterstaining the cell nuclei with hematoxylin. Finally, the slides were dehydrated, mounted, and observed under a microscope for image acquisition. The F4/80 positive area was counted using Image J software.

### 2.10 Prediction of a hub Gene-miRNAs network

The Hub genes selected after *in vivo* experiments were further input into the miRWalk database to predict potential miRNA regulatory networks. The results obtained were visualized using Cytoscape 3.9.1.

### 2.11 Statistical analysis

All data analysis was performed using GraphPad Prism 7 and the corresponding R packages. Experimental data are presented as mean ± standard deviation. An unpaired Student’s t-test was used to compare continuous variables between groups. A p-value less than 0.05 was considered statistically significant.

## 3 Results

### 3.1 DEGs in ALI and functional enrichment analysis

**Figure 2:**
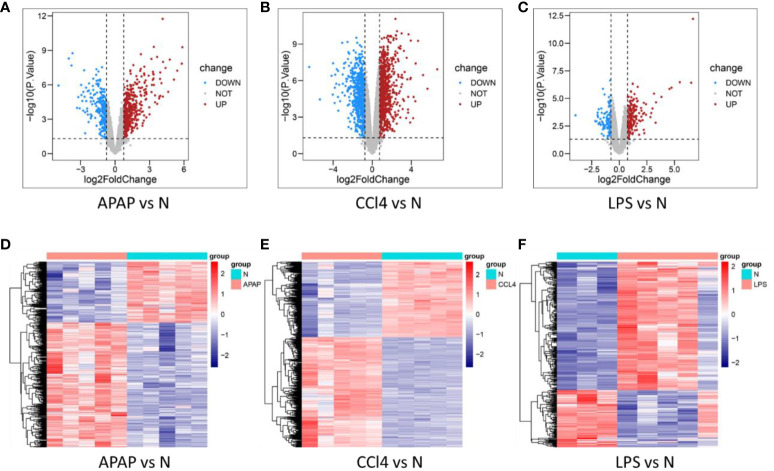
Determination of DEGs in three models. **(A–C)** volcano plot of DEGs in APAP, CCl4, and LPS. **(D–F)** Heat maps of DEGs in APAP, CCl4, and LPS.

Differential expression analysis results showed 799 DEGs in GSE167032, with 537 upregulated and 262 downregulated. In GSE167033, there were 1872 DEGs, including 1110 upregulated and 762 downregulated. Additionally, GSE166488 had 340 DEGs, with 235 upregulated and 105 downregulated. The differential analysis results were visualized as volcano plots and heatmaps (**Figure 2**).

**Figure 3:**
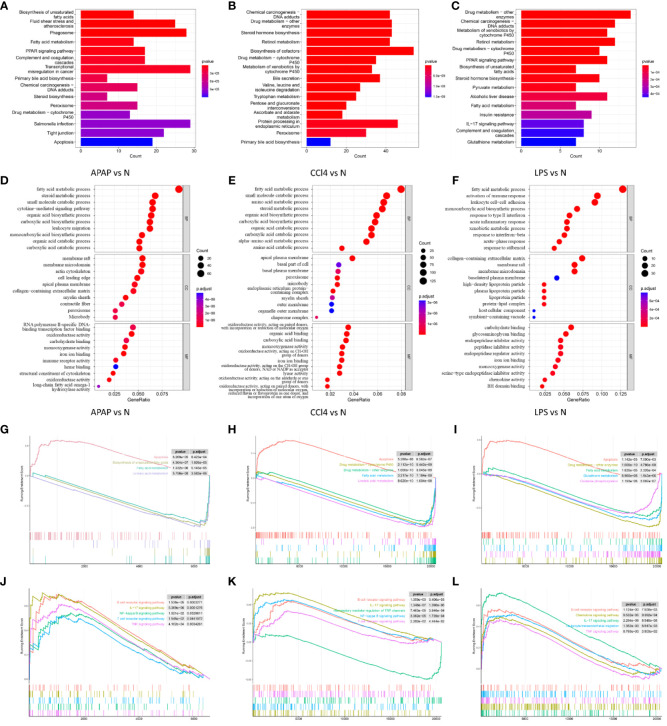
Functional enrichment analysis. **(A–C)** KEGG analysis of DEGs in APAP, CCl4, and LPS. **(D–F)** The top 10 functional enrichment in BP, CC, and MF analysis of APAP, CCl4, and LPS. **(G–I)** GSEA of apoptosis, fatty acid metabolism, and drug metabolism-related pathways in APAP, CCl4, and LPS. **(J–L)** GSEA sets of inflammatory response and immune response-related pathways in APAP, CCl4, and LPS.

KEGG enrichment analysis reveals that ALI induced by APAP, CCl4, and LPS primarily involves fatty acid metabolism, drug metabolism, PPAR signaling pathway, and IL-17 signaling pathway (**Figures 3A–C**).

The GO analysis results reveal that ALI is significantly associated with the GO terms in biological process (BP), cellular component (CC), and molecular function (MF). The enriched GO terms include fatty acid metabolism, inflammatory response, immune response, oxidation-reduction, and membrane microdomain, etc (**Figures 3D–F**).

GSEA analysis suggests that ALI induced by APAP, CCl4, or LPS is not only associated with biological processes such as apoptosis, fatty acid metabolism, and drug metabolism but is also closely related to inflammatory and immune-related pathways including the TNF signaling pathway, NF-kappa B signaling pathway, IL-17 signaling pathway, B cell receptor signaling pathway, and T cell receptor signaling pathway (**Figures 3G–L**).

### 3.2 PPI network analysis and hub DEGs identification

**Figure 4:**
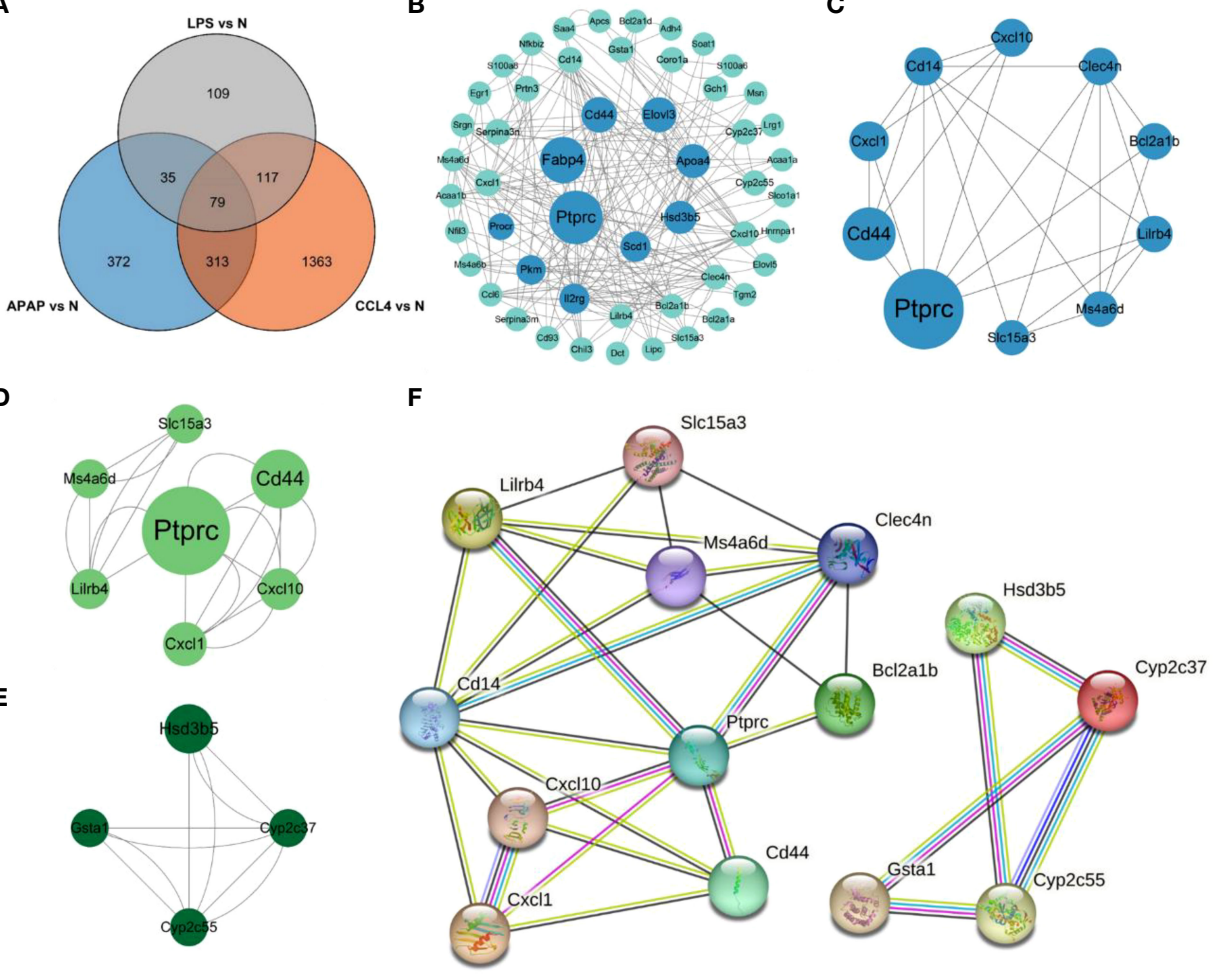
PPI network analysis and Hub gene identification. **(A)** Venn diagram shows the overlap of DEGs in APAP, CCl4 and LPS. **(B)** PPI network maps about 79 overlapping genes. The light green color represents the top 10 genes arranged according to betweenness. **(C)** Network diagram about the top10 DEGs of CytoHubba predictions. **(D, E)** Two clusters predicted by MCODE. **(F)** String analysis network diagram of 14 hub genes obtained by combining CytoHubba and MCODE algorithms.

Integrating the DEGs from the three datasets yielded 79 overlapping genes, with 51 being upregulated and 21 being downregulated (**Figure 4A**). The 79 overlapping genes were subjected to network analysis using the String database, and the results were visualized using Cytoscape (**Figure 4B**). As indicated in the red nodes in **Figure 4C**, the top 10 ranked hub genes obtained using the MCC algorithm from the CytoHubba plugin were Ptprc, Cd14, Clec4n, Ms4a6d, Cxcl10, Cd44, Lilrb4, Cxcl1, Slc15a3 and Bcl2a1b. According to the MCODE plugin analysis, two significant gene clusters were identified in **Figures 4D, E**. They were referred to as Cluster1, which includes the genes Cyp2c37, Gsta1, Hsd3b5, and Cyp2c55, and Cluster2, which consists of the genes Lilrb4, Ptprc, Slc15a3, Cd44, Ms4a6d, Cxcl10, and Cxcl1. Combining the results of the two analysis methods, 14 potential hub genes associated with ALI were determined. These genes include Ptprc, Cd14, Clec4n, Ms4a6d, Cxcl10, Cd44, Lilrb4, Cxcl1, Bcl2a1b, Cyp2c37, Gsta1, Hsd3b5, Cyp2c55, and Slc15a3. Subsequently, the 14 identified hub genes were input into the String database to obtain the PPI network graph shown in **Figure 4F**.

### 3.3 Immune cell infiltration in ALI

**Figure 5:**
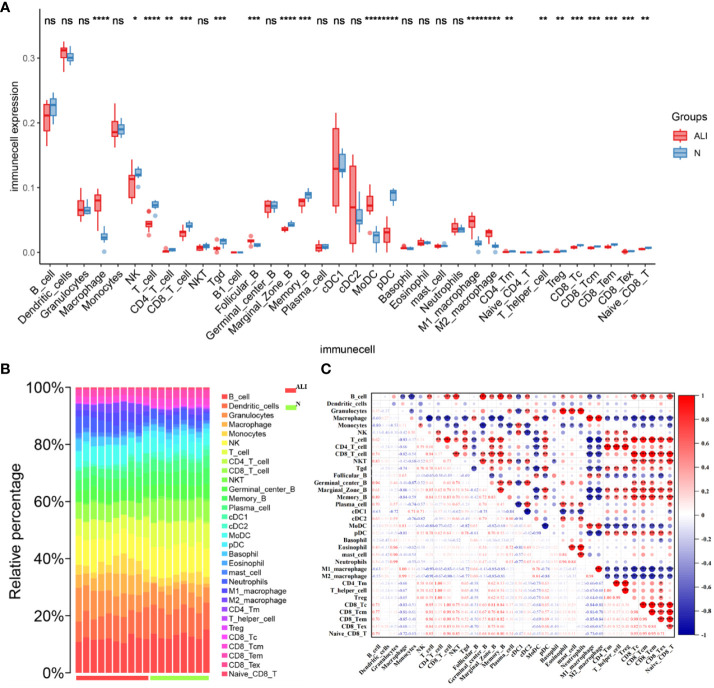
Characteristics of immune cell infiltration in ALI model. **(A)** Immune cell infiltration in ALI group and control group. **(B)** Stacked bar chart of immune cell infiltration in ALI group and control group. **(C)** The result of immune cell correlation analysis. *p<0.1, **p<0.05, ***p<0.01, ****p<0.001, ns, not significant.

After conducting immunoinfiltration analysis using the ImmuCell AI algorithm on the integrated results of GSE167033, GSE166488, and their respective datasets, significant differences in immune cell infiltration were observed between the ALI model and the control group. As displayed in **Figures 5A, B** and **Figure S1**, there is a significant increase in the infiltration of Macrophage, M1 Macrophage, and M2 Macrophage in the ALI model compared to the control group (p < 0.05). Conversely, Marginal Zone B, Memory B, and pDC infiltration decreased (p < 0.05). Further estimations were conducted on the correlation between immune cells, and the results showed a significant positive correlation between Macrophage, M1 Macrophage, and M2 Macrophage, as well as CD4 T cell, CD4 Tm, T helper cell, and Treg (**Figure 5C**). T cells negatively correlated with macrophage cells, M1 Macrophage, and M2 Macrophage.

### 3.5 Relationship between hub DEGs and immune cells

**Figure 6:**
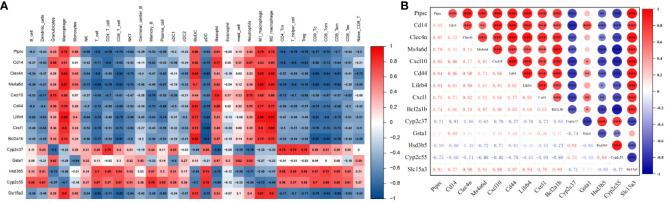
Relationship between hub genes and immune cells. **(A)** Correlation between hub genes and immune cells. **(B)** Correlation analysis of hub genes expression. *p<0.05, **p<0.01, ***p<0.001.

The potential correlation between immune cells and hub genes was analyzed. The results, as displayed in **Figure 6A**, indicate that hub genes exhibit contrasting correlations between macrophages and DC cells, T cells. Immunoinfiltration analysis determined a close association between macrophages and the ALI model. Focusing on macrophage analysis, there is a strong correlation between Clec4n (0.84), Ms4a6d (0.87), Lilrb4 (0.88), and macrophages, indicating their research significance in the context of macrophage infiltration-related ALI models. Through correlation analysis of hub genes, it was found that Clec4n exhibits strong correlations with Ptprc (0.87), Cd44 (0.90), Lilrb4 (0.88), Cxcl1 (0.82), Slc15a3 (0.90); Ms4a6d was associated with Cxcl10 (0.81), Bcl2a1b (0.87), Cyp2c55 (-0.88), Slc15a3 (0.81); and Lilrb4 was strongly correlated with Ptprc (0.84), Cd14 (0.81), Clec4n (0.88), Cxcl10 (0.81), Cd44 (0.87), Cxcl1 (0.82), Slc15a3 (0.84) (**Figure 6B**).

### 3.6 Assessment of ALI and macrophage infiltration in validated models

**Figure 7:**
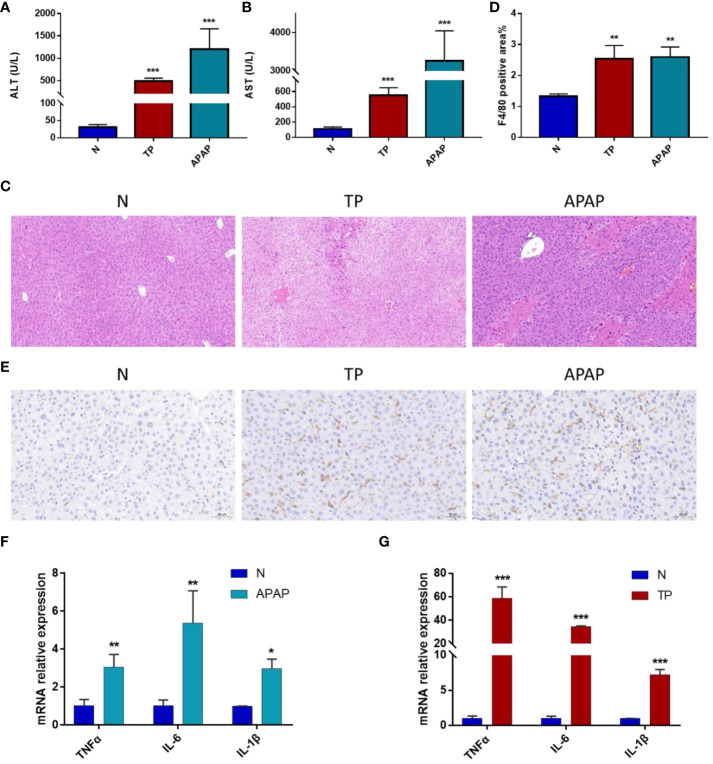
Validation of ALI model induced by APAP and TP. **(A)** ALT levels in serum. **(B)** AST levels in serum. **(C)** HE staining results of liver tissue. Scale bars: 50 μm. **(D)** Quantification of macrophage infiltration. **(E)** Immunohistochemical results. Scale bars: 50 μm. **(F)** mRNA expression of TNF-α, IL-6 and IL-1β in liver tissue of APAP model. **(G)** mRNA expression of TNF-α, IL-6 and IL-1β in liver tissue of TP model. *p<0.05, **p<0.01, ***p<0.001 APAP/TP vs N.

Based on the above results, the APAP and TP-induced ALI models were selected to validate the relationship between hub genes, macrophage infiltration, and ALI. In the validated model of ALI, a significant increase in serum AST and ALT levels was observed after treatment with TP and APAP (**Figures 7A, B**). HE staining revealed a pronounced occurrence of hepatocyte steatosis in the mouse liver tissue following TP intervention, accompanied by hepatocyte swelling, hepatocyte necrosis, and localized infiltration of lymphocytes. In the APAP-induced ALI model, a substantial extent of hepatocyte necrosis was observed, accompanied by increased fibroblast proliferation, hepatocyte steatosis, and infiltration of lymphocytes and granulocytes (**Figure 7C**). Based on the analysis of immune infiltration, macrophage infiltration exhibited an increasing trend in all three models of ALI. Consequently, F4/80 staining was utilized to observe the distribution of macrophages in the TP and APAP-induced ALI models (8). It was found that compared to the control group, macrophage infiltration significantly increased in the TP and APAP groups, consistent with the previous analysis of immune infiltration (**Figures 7D, E**). Furthermore, the expression of macrophage-related inflammatory factors TNFα, IL-6, and IL-1β in liver tissue was evaluated. The results were as expected, with a significant upregulation of TNFα, IL-6, and IL-1β expression observed in the TP and APAP-induced ALI model (**Figures 7F, G**).

### 3.7 Experimental validations of hub DEGs expression in ALI mouse

**Figure 8:**
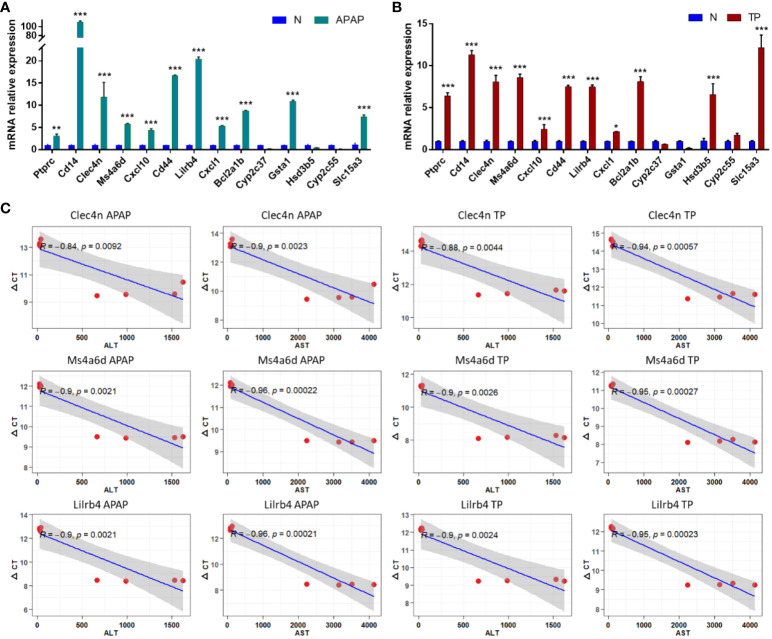
Hub DEGs expression in ALI mouse. **(A, B)** Validation of hub gene expression in APAP and TP models. **(C)** The correlation between Clec4n, Ms4a6d, Lilrb and liver function indicators ALT and AST. *p<0.05, **p<0.01, ***p<0.001 APAP/TP vs N.

Liver tissue RNA was extracted from mice with APAP- and TP-induced ALI to evaluate the expression levels of 14 hub genes using RT-qPCR. The results, as depicted in **Figures 8A, B**, demonstrate significant differences in the expression levels of Ptprc, Cd14, Clec4n, Ms4a6d, Cxcl10, Cd44, Lilrb4, Cxcl1, Bcl2a1b, and Slc15a3 in both validation models of ALI, exhibiting the expected trend. Moreover, the correlation between Clec4n, Ms4a6d, Lilrb4, and ALT, AST was analyzed (**Figure 8C**). The results revealed a strong correlation (p<0.01) between the PCR cycle numbers of Clec4n in both the APAP and TP models and ALT (-0.84, -0.88) and AST (-0.9, -0.94). Similarly, the PCR cycle numbers of Ms4a6d in the APAP and TP models showed a high correlation (p<0.01) with ALT (-0.9, -0.9) and AST (-0.96, -0.95). Furthermore, Lilrb4 displayed a significant correlation (p<0.01) with ALT (-0.9, -0.9) and AST (-0.96, -0.95) in both the APAP and TP models.

### 3.9 Prediction of hub gene and miRNA networks

**Figure 9:**
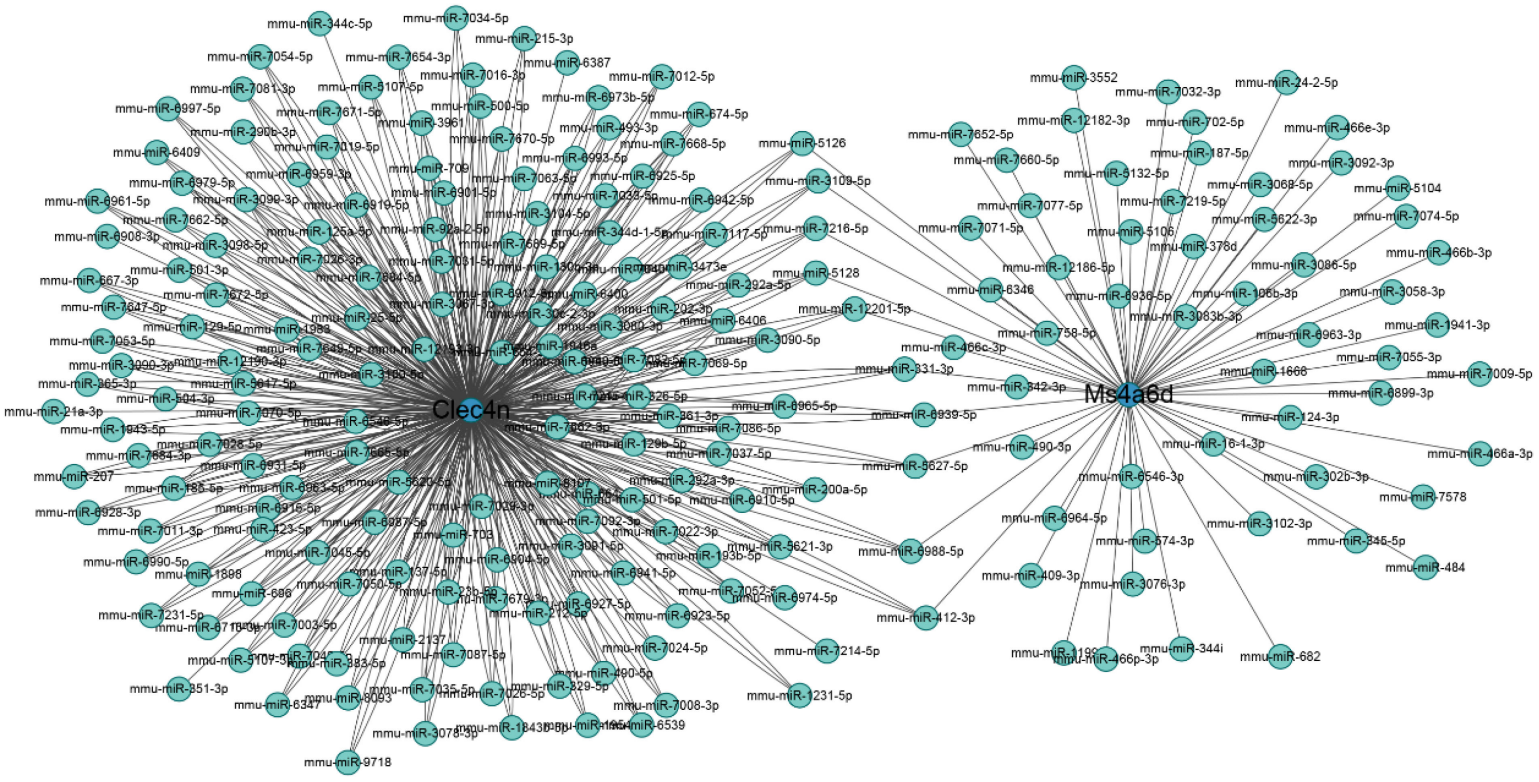
Regulatory networks on miRNA and macrophage-related genes. Blue circles represent genes, and cyan dots represent miRNA.

The miRNA regulatory networks of the hub genes Clec4n, Ms4a6d, and Lilrb4 were predicted using the miRWalk database. The results only included the miRNA regulatory network associated with Clec4n and Ms4a6d, which comprised 223 nodes and 562 edges (**Figure 9**). Notably, miR-5126, miR-3109-5p, miR-7216-5p, miR-5128, miR-12201-5p, miR331-3p, miR-6939-5p, miR-5627-5p, miR-6988-5p, and miR-412-3p, were found to interact with the hub genes Clec4n and Ms4a6d. These results highlight the potential research value of these miRNAs in the context of ALI. However, it is important to note that further validation is required to establish their roles fully.

## 4 Discussion

The liver is an essential organ in the human body, performing a wide range of vital physiological functions. It plays a crucial role in metabolizing nutrients, detoxifying harmful substances, producing bile for digestion, storing vitamins and minerals, and synthesizing proteins and clotting factors. However, the liver is susceptible to various forms of injury, including ALI. ALI refers to the sudden and severe damage to liver cells, which can be caused by various factors such as viral infections, drug toxicity, alcohol abuse, or autoimmune reactions. When the liver experiences acute injury, its normal physiological functions can be significantly impaired. Understanding general mechanisms and regulatory targets is crucial for comprehending the ALI process and identifying potential therapeutic drugs. In this study, APAP, CCl4, and LPS were selected as the research subjects for ALI. These agents are commonly used in animal experiments to induce ALI and involve multiple mechanisms, including inflammation response, oxidative stress, immune response, apoptosis, and necrosis (1, 2, 5, 16). By employing various bioinformatics methods, this study screened and identified a set of common DEGs from three models. Through the predictions of MCODE and CytoHubba, a potential set of hub genes was identified. Functional enrichment analysis revealed that the three ALI datasets were primarily associated with pathways related to fatty acid metabolism, drug metabolism, inflammatory response, and immune response. Additionally, the infiltration characteristics of immune cells in different models were predicted, and the correlation between hub genes and immune cells was also examined. Finally, the immune infiltration and target gene expression results were validated by combining the APAP and TP-induced ALI models *in vivo*. By incorporating multiple models of ALI, this study may provide insights into the general regulatory mechanisms of ALI and offer references for exploring general solutions to ALI.

This study is the first attempt to combine three commonly used models of ALI to explore the general mechanisms underlying the occurrence of ALI. PPI network analysis identified 14 DEGs potentially connected to ALI. Through validation using a mouse model of ALI, a final selection of 10 genes (Ptprc, Cd14, Clec4n, Ms4a6d, Cxcl10, Cd44, Lilrb4, Cxcl1, Bcl2a1b, and Slc15a3) was confirmed to be significantly associated with ALI. These 10 hub genes have been reported to play regulatory roles in immune regulation and inflammatory response. For example, Ptprc, as a transmembrane glycoprotein, is an essential regulator of T cell and B cell antigen receptor-mediated activation. It is essential in immune deficiency, autoimmune diseases or malignant tumors (30). Due to its regulatory role in the immune response signaling pathway, Ptprc is also used as a target for treating certain immune diseases, such as organ transplant rejection and tumor metastasis, by selectively inhibiting Ptprc (31, 32). Bcl2a1b is associated with neutrophil proliferation and can serve as a potential marker for identifying proliferating neutrophils, aiding in the study and clinical identification and characterization of neutrophils in ALI. The accumulation of neutrophils contributes to the production of CCL6, thereby recruiting more macrophages (33, 34). Slc15a3 is highly expressed in macrophages and plays a vital role in inflammatory diseases. Studies have shown that knocking down Slc15a3 can reduce the production of TLR4-dependent TNF-α and IL-6. Furthermore, the expression of Slc15a3 positively correlates with the inflammatory process, indicating that Slc15a3 has value for the treatment and evaluation of inflammatory diseases (35, 36). Cxcl10 plays a crucial role in recruiting immune cells during the liver infiltration process in liver diseases. It has been confirmed that Cxcl10 is necessary for promoting inflammatory responses and can participate in hepatocyte apoptosis and inflammatory reactions through toll-like receptor 4 (37, 38). Cxcl1 has been reported to be associated with the recruitment of neutrophils and the severity of liver disease during liver injury. The detection of Cxcl1 levels may have clinical significance in assessing the progression of ALI (39, 40).

ALI is closely linked to dysfunction in innate immunity. GSEA analysis has revealed that all three ALI models are associated with signaling pathways related to inflammation, immune response, and apoptosis (2, 41). In this study, by comparing different immune cell infiltration in the ALI model and the control group, it was found that macrophage infiltration was significantly increased in the ALI model. Immunohistochemical staining showed that the distribution of macrophages in the TP and APAP-induced ALI models was significantly up-regulated. Macrophages are the most abundant immune cells in the liver and play a pivotal role in maintaining liver homeostasis and the potential mechanisms underlying liver diseases (42). In ALI, activated macrophages can increase the release of inflammatory cytokines (IL-6, TNF-α), nitrogen species, reactive oxygen species, and other factors, activating apoptotic pathways in liver cells. Furthermore, macrophage derived chemokines (IL-18, MCP-1) further recruit other immune cells to intervene in the process of liver injury (2, 13–15). Macrophage infiltration in the liver is a hallmark and contributor to liver inflammation and injury (43). Animal experiments have demonstrated that depleting of macrophages can effectively improve APAP-induced liver injury (44). Promoting the transition of macrophage functional characteristics from pro-inflammatory to anti-inflammatory can facilitate the resolution of tissue damage responses (45). Inhibiting macrophage migration to suppress pro-inflammatory immune activation can protect against ischemic liver injury (46). The transition of macrophage phenotypes from pro-inflammatory to anti-inflammatory can be regulated through various biological mechanisms, such as JAK/STATs, TLR4/NF-kB, PI3K/Akt/mTOR, PPARγ, TGF-β/Smads pathways, among others. Targeting these pathways involved in macrophage phenotype transition is a feasible strategy to alleviate ALI (47). For example, studies have found that reducing the expression of HIF-1α and activating the PI3K/Akt/mTOR signaling pathway can decrease oxidative stress and regulate macrophage polarization in the liver, thereby alleviating injury (48).

Integrating the results of hub gene and immune infiltration showed that Clec4n, Ms4a6d and Lilrb4 were strongly correlated with macrophages and serum ALT and AST levels. These results suggest that Clec4n, Ms4a6d, and Lilrb4 are associated with macrophage infiltration in ALI. Clec4n is a C-type lectin receptor expressed on dendritic cells and macrophages (49). Studies have shown that knocking down Clec4n in macrophages significantly affects the secretion of TNF-α, IL-6, or MIP-2 (50). Recent research has demonstrated that in acute inflammation, Ms4a6d in macrophages promotes the transcription of pro-inflammatory genes and increases mitochondrial reactive oxygen species secretion through crosslinking with MHC Class II antigen (51). Lilrb4 is expressed on the surface of antigen-presenting cells such as macrophages and dendritic cells (52). Studies have found that Lilrb4 is involved in the pathological processes of various inflammatory diseases, and downregulation of Lilrb4 exacerbates local inflammatory responses (53). Knockout Lilrb4 in mice results in increased secretion of pro-inflammatory cytokines TNF-α and IL-6, and silencing Lilrb4 *in vitro* also leads to increased production of pro-inflammatory cytokines IL-6 and IL-1β (54, 55). These findings suggest that downregulation of Lilrb4 exacerbates the inflammatory process, and targeting Lilrb4 or its associated pathways may be an effective strategy for mitigating inflammation-induced injury in ALI.

However, there are some limitations in this study. For instance, the three ALI models used may not represent all types of ALI. The sample size was not sufficiently large, and the potential regulatory targets obtained were not validated in a more significant number of ALI models. Nevertheless, it is hoped that this study can lay the foundation for exploring the general regulatory targets and mechanisms of ALI, providing insights for clinical strategies to address ALI.

## 5 Conclusion

In summary, this study employed three datasets of ALI induced by APAP, CCl4, and LPS as generic models for liver injury research. A total of 79 overlapping genes were identified, identifying 14 potential hub genes associated with ALI. Functional enrichment analysis indicated that the ALI process may be linked to pathways related to fatty acid metabolism, drug metabolism, inflammatory response, and immune response. Immunoinfiltration studies revealed a significant increase in macrophage infiltration in ALI models. By constructing APAP and TP-induced ALI models, we validated the regulation of the 10 hub genes at the *in vivo* level, confirming consistency with the bioinformatics analysis results. Correlation analysis identified Clec4n, Ms4a6d, and Lilrb4 as significantly correlated with macrophage infiltration and liver function biochemical markers ALT and AST, suggesting their potential for further investigation in the prevention and treatment of ALI. Furthermore, using the miRWalk database, 10 miRNAs with potential research value in ALI were predicted.

## Data availability statement

The original contributions presented in the study are included in the article/**Supplementary Material**. Further inquiries can be directed to the corresponding author.

## Ethics statement

The experimental protocol was approved by the Research Ethics Committee of the Institute of Basic Theory of Chinese Medicine, China Academy of Chinese Medical Sciences (IBTCMCACMS21-2110-04). The study was conducted in accordance with the local legislation and institutional requirements.

## Author contributions

ZC: Writing – original draft, Writing – review & editing. PL: Data curation, Formal analysis, Writing – review & editing. LL (3^rd^ author): Conceptualization, Data curation, Writing – original draft. QG: Methodology, Visualization, Writing – original draft. LLin: Methodology, Writing – original draft. LY: Validation, Visualization, Writing – original draft. LZ: Validation, Writing – original draft. CS: Validation, Writing – original draft. LL (9^th^ author): Writing – review & editing. NZ: Writing – review & editing. XH: Writing – review & editing. YT: Writing – review & editing. CL: Writing – review & editing.

## References

[1] Alkandahri MY Pamungkas BT Oktoba Z Shafirany MZ Sulastri L Arfania M. Hepatoprotective effect of kaempferol: A review of the dietary sources, bioavailability, mechanisms of action, and safety. Adv Pharmacol Pharm Sci (2023) 2023:1–16. doi: 10.1155/2023/1387665 PMC9988374 36891541

[2] Yang Y Ni M Zong R Yu M Sun Y Li J. Targeting notch1-yap circuit reprograms macrophage polarization and alleviates acute liver injury in mice. Cell Mol Gastroenterol Hepatol (2023) 15(5):1085–104. doi: 10.1016/j.jcmgh.2023.01.002 PMC10036742 36706917

[3] Qin C-C Liu Y-N Hu Y Yang Y Chen Z. Macrophage inflammatory protein-2 as mediator of inflammation in acute liver injury. World J Gastroenterol (2017) 23(17):3043–52. doi: 10.3748/wjg.v23.i17.3043 PMC5423041 28533661

[4] Roth K Strickland J Copple BL. Regulation of macrophage activation in the liver after acute injury: role of the fibrinolytic system. World J Gastroenterol (2020) 26(16):1879–87. doi: 10.3748/wjg.v26.i16.1879 PMC7201151 32390699

[5] Frank D Savir S Gruenbaum B Melamed I Grinshpun J Kuts R. Inducing acute liver injury in rats via carbon tetrachloride (Ccl4) exposure through an orogastric tube. J visualized experiments JoVE (2020) 158:e60695. doi: 10.3791/60695 PMC7859859 32420997

[6] Yu C Chen P Miao L Di G. The role of the nlrp3 inflammasome and programmed cell death in acute liver injury. Int J Mol Sci (2023) 24(4):3067. doi: 10.3390/ijms24043067 36834481 PMC9959699

[7] Hirao H Nakamura K Kupiec-Weglinski J. Liver ischaemia-reperfusion injury: A new understanding of the role of innate immunity. Nat Rev Gastroenterol Hepatol (2022) 19(4):239–56. doi: 10.1038/s41575-021-00549-8 34837066

[8] Xie Y Zhong K-B Hu Y Xi Y-L Guan S-X Xu M. Liver infiltration of multiple immune cells during the process of acute liver injury and repair. World J Gastroenterol (2022) 28(46):6537–50. doi: 10.3748/wjg.v28.i46.6537 PMC9782841 36569272

[9] Khoury T Rmeileh A Yosha L Benson A Daher S Mizrahi M. Drug induced liver injury: review with a focus on genetic factors, tissue diagnosis, and treatment options. J Clin Trans Hepatol (2015) 3(2):99–108. doi: 10.14218/jcth.2015.00007 PMC4548351 26356634

[10] Bernal W Auzinger G Dhawan A Wendon J. Acute liver failure. Lancet (London England) (2010) 376(9736):190–201. doi: 10.1016/s0140-6736(10)60274-7 20638564

[11] Karlmark K Wasmuth H Trautwein C Tacke F. Chemokine-directed immune cell infiltration in acute and chronic liver disease. Expert Rev Gastroenterol Hepatol (2008) 2(2):233–42. doi: 10.1586/17474124.2.2.233 19072358

[12] Ayata C Ganal S Hockenjos B Willim K Vieira R Grimm M. Purinergic P2y_2_ Receptors promote neutrophil infiltration and hepatocyte death in mice with acute liver injury. Gastroenterology (2012) 143(6):1620–9.e4. doi: 10.1053/j.gastro.2012.08.049 22974709

[13] Brenner C Galluzzi L Kepp O Kroemer G. Decoding cell death signals in liver inflammation. J Hepatol (2013) 59(3):583–94. doi: 10.1016/j.jhep.2013.03.033 23567086

[14] Ilyas G Zhao E Liu K Lin Y Tesfa L Tanaka K. Macrophage autophagy limits acute toxic liver injury in mice through down regulation of interleukin-1β. J Hepatol (2016) 64(1):118–27. doi: 10.1016/j.jhep.2015.08.019 PMC4691423 26325539

[15] Xu L Yang Y Wen Y Jeong J-M Emontzpohl C Atkins CL. Hepatic recruitment of eosinophils and their protective function during acute liver injury. J Hepatol (2022) 77(2):344–52. doi: 10.1016/j.jhep.2022.02.024 PMC9308653 35259470

[16] Mohanraj R Yao L Chen W Song K Han C Gandhi CR. 15-hydroxyprostaglandin dehydrogenase (15-pgdh) prevents lipopolysaccharide (Lps)-induced acute liver injury. PloS One (2017) 12(4):e0176106. doi: 10.1371/journal.pone.0176106 28423012 PMC5397067

[17] Yan M Huo Y Yin S Hu H. Mechanisms of acetaminophen-induced liver injury and its implications for therapeutic interventions. Redox Biol (2018) 17:274–83. doi: 10.1016/j.redox.2018.04.019 PMC6006912 29753208

[18] Zhang H Yuan Z Zhu Y Yuan Z Wang J Nong C. Th17/treg imbalance mediates hepatic intolerance to exogenous lipopolysaccharide and exacerbates liver injury in triptolide induced excessive immune response. J Ethnopharmacology (2022) 295:115422. doi: 10.1016/j.jep.2022.115422 35654348

[19] Wang X Sun L Zhang L Jiang Z. Effect of adoptive transfer or depletion of regulatory T cells on triptolide-induced liver injury. Front Pharmacol (2016) 7:99. doi: 10.3389/fphar.2016.00099 27148057 PMC4840269

[20] Feng Z Zhou C Dong S Liu Z Liu T Zhou L. Catalpol and panax notoginseng saponins synergistically alleviate triptolide-induced hepatotoxicity through nrf2/are pathway. Toxicol In Vitro (2019) 56:141–9. doi: 10.1016/j.tiv.2019.01.016 30682494

[21] Holland CH Ramirez Flores RO Myllys M Hassan R Edlund K Hofmann U. Transcriptomic cross-species analysis of chronic liver disease reveals consistent regulation between humans and mice. Hepatol Commun (2021) 6(1):161–77. doi: 10.1002/hep4.1797 PMC8710791 34558834

[22] Ritchie ME Phipson B Wu D Hu Y Law CW Shi W. Limma powers differential expression analyses for rna-sequencing and microarray studies. Nucleic Acids Res (2015) 43(7):e47–e. doi: 10.1093/nar/gkv007 PMC4402510 25605792

[23] Gustavsson E Zhang D Reynolds R Garcia-Ruiz S Ryten M. Ggtranscript: an R package for the visualization and interpretation of transcript isoforms using ggplot2. Bioinf (Oxford England) (2022) 38(15):3844–6. doi: 10.1093/bioinformatics/btac409 PMC9344834 35751589

[24] Wu T Hu E Xu S Chen M Guo P Dai Z. Clusterprofiler 4.0: A universal enrichment tool for interpreting omics data. Innovation (2021) 2(3):100141. doi: 10.1016/j.xinn.2021.100141 34557778 PMC8454663

[25] Shannon P Markiel A Ozier O Baliga NS Wang JT Ramage D. Cytoscape: A software environment for integrated models of biomolecular interaction networks. Genome Res (2003) 13(11):2498–504. doi: 10.1101/gr.1239303 PMC403769 14597658

[26] Bader G Hogue C. An automated method for finding molecular complexes in large protein interaction networks. BMC Bioinf (2003) 4:2. doi: 10.1186/1471-2105-4-2 PMC149346 12525261

[27] Chin C-H Chen S-H Wu H-H Ho C-W Ko M-T Lin C-Y. Cytohubba: identifying hub objects and sub-networks from complex interactome. BMC Syst Biol (2014) 8(S4):S11. doi: 10.1186/1752-0509-8-s4-s11 25521941 PMC4290687

[28] Miao Y-R Xia M Luo M Luo T Yang M Guo A-Y. Immucellai-mouse: A tool for comprehensive prediction of mouse immune cell abundance and immune microenvironment depiction. Bioinformatics (2022) 38(3):785–91. doi: 10.1093/bioinformatics/btab711 34636837

[29] Miao YR Zhang Q Lei Q Luo M Xie GY Wang H. Immucellai: A unique method for comprehensive T-cell subsets abundance prediction and its application in cancer immunotherapy. Advanced Sci (2020) 7(7):1902880. doi: 10.1002/advs.201902880 PMC7141005 32274301

[30] Al Barashdi MA Ali A McMullin MF Mills K. Protein tyrosine phosphatase receptor type C (Ptprc or cd45). J Clin Pathol (2021) 74(9):548–52. doi: 10.1136/jclinpath-2020-206927 PMC8380896 34039664

[31] Urbanek R Suchard S Steelman G Knappenberger K Sygowski L Veale C. Potent reversible inhibitors of the protein tyrosine phosphatase cd45. J medicinal Chem (2001) 44(11):1777–93. doi: 10.1021/jm000447i 11356112

[32] Perron M Saragovi H. Inhibition of cd45 phosphatase activity induces cell cycle arrest and apoptosis of cd45 lymphoid tumors ex vivo and in vivo. Mol Pharmacol (2018) 93(6):575–80. doi: 10.1124/mol.117.110908 29555821

[33] Liu Y Zhang M Liao Y Chen H Su D Tao Y. Human umbilical cord mesenchymal stem cell-derived exosomes promote murine skin wound healing by neutrophil and macrophage modulations revealed by single-cell rna sequencing. Front Immunol (2023) 14:1142088. doi: 10.3389/fimmu.2023.1142088 36999022 PMC10044346

[34] Cowburn A Summers C Dunmore B Farahi N Hayhoe R Print C. Granulocyte/macrophage colony-stimulating factor causes a paradoxical increase in the bh3-only pro-apoptotic protein bim in human neutrophils. Am J Respir Cell Mol Biol (2011) 44(6):879–87. doi: 10.1165/rcmb.2010-0101OC PMC4373550 20705940

[35] Song F Yi Y Li C Hu Y Wang J Smith DE. Regulation and biological role of the peptide/histidine transporter slc15a3 in toll-like receptor-mediated inflammatory responses in macrophage. Cell Death Dis (2018) 9(7):770. doi: 10.1038/s41419-018-0809-1 29991810 PMC6039463

[36] Wang Y Hu Y Li P Weng Y Kamada N Jiang H. Expression and regulation of proton-coupled oligopeptide transporters in colonic tissue and immune cells of mice. Biochem Pharmacol (2018) 148:163–73. doi: 10.1016/j.bcp.2017.12.025 PMC5801143 29305856

[37] Liu H Ling CC Yeung WHO Pang L Liu J Zhou J. Monocytic mdsc mobilization promotes tumor recurrence after liver transplantation via cxcl10/tlr4/mmp14 signaling. Cell Death Dis (2021) 12(5):489. doi: 10.1038/s41419-021-03788-4 33990548 PMC8121858

[38] Hintermann E Bayer M Pfeilschifter J Luster A Christen U. Cxcl10 promotes liver fibrosis by prevention of nk cell mediated hepatic stellate cell inactivation. J Autoimmun (2010) 35(4):424–35. doi: 10.1016/j.jaut.2010.09.003 PMC3855675 20932719

[39] Chang B Xu MJ Zhou Z Cai Y Li M Wang W. Short- or long-term high-fat diet feeding plus acute ethanol binge synergistically induce acute liver injury in mice: an important role for cxcl1. Hepatology (2015) 62(4):1070–85. doi: 10.1002/hep.27921 PMC4589443 26033752

[40] Dominguez M Miquel R Colmenero J Moreno M García-Pagán J Bosch J. Hepatic expression of cxc chemokines predicts portal hypertension and survival in patients with alcoholic hepatitis. Gastroenterology (2009) 136(5):1639–50. doi: 10.1053/j.gastro.2009.01.056 19208360

[41] Bernal W Lee W Wendon J Larsen F Williams R. Acute liver failure: A curable disease by 2024? J Hepatol (2015) 62:S112–20. doi: 10.1016/j.jhep.2014.12.016 25920080

[42] Krenkel O Tacke F. Liver macrophages in tissue homeostasis and disease. Nat Rev Immunol (2017) 17(5):306–21. doi: 10.1038/nri.2017.11 28317925

[43] Liu Z Wang M Wang X Bu Q Wang Q Su W. Xbp1 deficiency promotes hepatocyte pyroptosis by impairing mitophagy to activate mtdna-cgas-sting signaling in macrophages during acute liver injury. Redox Biol (2022) 52:102305. doi: 10.1016/j.redox.2022.102305 35367811 PMC8971356

[44] TangCCenLZengHZhangXLiuPChenY. Inhibiting hepatocyte uric acid synthesis and reabsorption ameliorates acetaminophen-induced acute liver injury in mice. Cell Mol Gastroenterol Hepatol (2023). doi: 10.1016/j.jcmgh.2023.10.005 37879407

[45] Arnold L Henry A Poron F Baba-Amer Y van Rooijen N Plonquet A. Inflammatory Monocytes Recruited after Skeletal Muscle Injury Switch into Antiinflammatory Macrophages to Support Myogenesis. J Exp Med (2007) 204(5):1057–69. doi: 10.1084/jem.20070075 PMC2118577 17485518

[46] Zhou H Zhou S Shi Y Wang Q Wei S Wang P. Tgr5/cathepsin E signaling regulates macrophage innate immune activation in liver ischemia and reperfusion injury. Am J Transplant Off J Am Soc Transplant Am Soc Transplant Surgeons (2021) 21(4):1453–64. doi: 10.1111/ajt.16327 32986275

[47] Wang C Ma C Gong L Guo Y Fu K Zhang Y. Macrophage polarization and its role in liver disease. Front Immunol (2021) 12:803037. doi: 10.3389/fimmu.2021.803037 34970275 PMC8712501

[48] Song B Zhang C Hu W Guo C Xia Z Hu W. Nano-Designed Carbon Monoxide Donor Sma/Corm2 Exhibits Protective Effect against Acetaminophen Induced Liver Injury through Macrophage Reprograming and Promoting Liver Regeneration. J Controlled release Off J Controlled Release Soc (2021) 331:350–63. doi: 10.1016/j.jconrel.2021.01.025 33482271

[49] Sun H Xu X Shao H Su X Wu X Wang Q. Dectin-2 is predominately macrophage restricted and exhibits conspicuous expression during aspergillus fumigatus invasion in human lung. Cell Immunol (2013) 284:60–7. doi: 10.1016/j.cellimm.2013.06.013 23928558

[50] Viriyakosol S MdP J Saijo S Fierer J Deepe GS. Neither dectin-2 nor the mannose receptor is required for resistance to coccidioides immitis in mice. Infection Immun (2014) 82(3):1147–56. doi: 10.1128/iai.01355-13 PMC3957980 24379281

[51] Chen Y Li S Huang X Wang C Pan Y Xiang Q. Tetraspan ms4a6d is a coreceptor of mhc class ii antigen (Mhc-ii) that promotes macrophages-derived inflammation. Mol Immunol (2023) 160:121–32. doi: 10.1016/j.molimm.2023.07.003 37429063

[52] Cella M Döhring C Samaridis J Dessing M Brockhaus M Lanzavecchia A. A novel inhibitory receptor (Ilt3) expressed on monocytes, macrophages, and dendritic cells involved in antigen processing. J Exp Med (1997) 185(10):1743–51. doi: 10.1084/jem.185.10.1743 PMC2196312 9151699

[53] Katz H. Inhibition of pathologic inflammation by leukocyte ig-like receptor B4 and related inhibitory receptors. Immunol Rev (2007) 217:222–30. doi: 10.1111/j.1600-065X.2007.00522.x 17498062

[54] Jiang Z Qin J-J Zhang Y Cheng W-L Ji Y-X Gong F-H. Lilrb4 deficiency aggravates the development of atherosclerosis and plaque instability by increasing the macrophage inflammatory response via nf-Kb signaling. Clin Sci (2017) 131(17):2275–88. doi: 10.1042/cs20170198 28743735

[55] Chang C Liu Z Vlad G Qin H Qiao X Mancini D. Ig-like transcript 3 regulates expression of proinflammatory cytokines and migration of activated T cells. J Immunol (Baltimore Md 1950) (2009) 182(9):5208–16. doi: 10.4049/jimmunol.0804048 19380766

