# Supplementary material for: Bioinformatics-led discovery of liver-specific genes and macrophage infiltration in acute liver injury
Source: Front Immunol. 2023 Dec 7;14:1287136. doi: 10.3389/fimmu.2023.1287136 (PMC10733525; doi:10.3389/fimmu.2023.1287136)
Supplement: Supplementary file 1 [file DataSheet_1.docx]

Table S1. PCR primers

| Gene | Forward primer | Reverse primer |
| --- | --- | --- |
| Ptprc | TTGACAGAGTTAGTGAATGGAGACC | AGTTCGGAGAGTGTAGGCTGAG |
| Cd14 | TGGCTTGTTGCTGTTGCTTCTG | ACCAATCTGGCTTCGGATCTGAG |
| Clec4n | TGGAGCACCAGTGAGCAGAAC | CCGAAAGACCCAGGAAGTAAGAAAG |
| Ms4a6d | GCTCTTACAGGCATCGCTATTCTC | TGGGTTGTGTCTAGTTGTGTGAAAG |
| Cxcl10 | GCCTCATCCTGCTGGGTCTG | TCATTCTCACTGGCCCGTCATC |
| Cd44 | CAAGTGCGAACCAGGACAGTG | CAGAGCCAGTGCCAGGAGAG |
| Lilrb4 | CTCTCTTGGACCCTGGACTCAC | AGTAGCCATAGCATCTGAATGTTCC |
| Cxcl1 | GCTGGGATTCACCTCAAGAACATC | GTGTGGCTATGACTTCGGTTTGG |
| Bcl2a1b | ACGGCGGAATGGAGGTTGG | CATTTCCCAGAACTGTCCTGTCATC |
| Cyp2c37 | TCACTCTCTCCTGTCTGTTTCTCC | ATTGGTGAAGGATTGGCAGATGTC |
| Gsta1 | GGCTGACCAGGGTGGACATC | GCTGCTGATTCTGCTCTTGAAGG |
| Hsd3b5 | CCACACCGCTGCTGCTATTG | AATGTTGGCACACTGGCTTCC |
| Cyp2c55 | GACAAAGAATTTCCCAACCCAGAAG | CACGCACATTCGCTTTCCTATTG |
| Slc15a3 | CGACGGTGGAGACGGACAG | TTGAGGTAGAGCACGAGGTTGG |


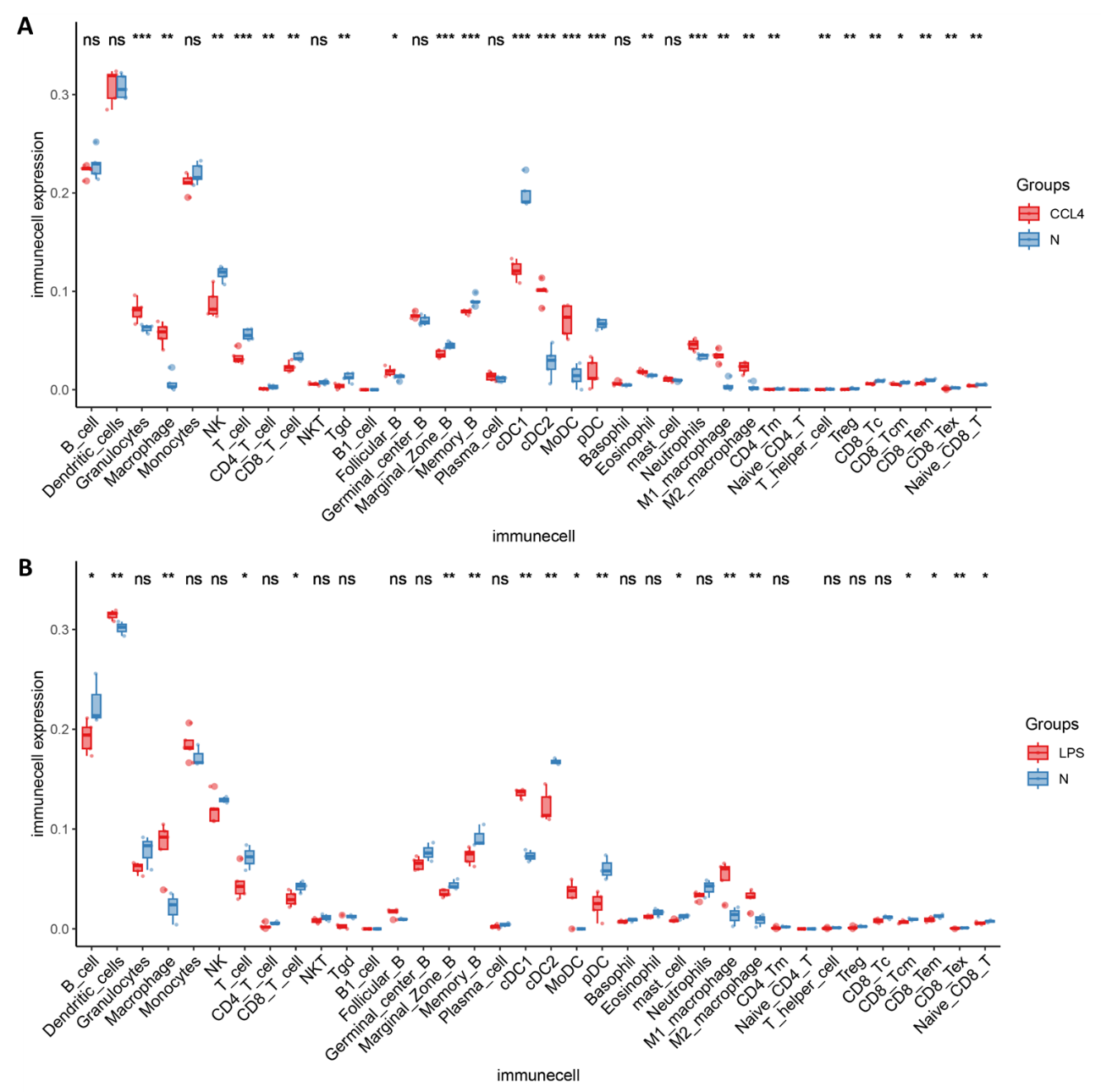


Figure S1. Immune cell infiltration in ALI group and control group. (A) Characteristics of immune infiltration in CCL4 model. (B) Characteristics of immune infiltration in LPS model.
